# Comparison of the effect of sevoflurane or propofol anesthesia on the regional cerebral oxygen saturation in patients undergoing carotid endarterectomy: a prospective, randomized controlled study

**DOI:** 10.1186/s12871-019-0820-9

**Published:** 2019-08-17

**Authors:** Sanghee Park, Keunbae Yook, Kyung Yeon Yoo, Jeong Il Choi, Hong-Beom Bae, Youngwook You, Baoyuan Jin, Seongtae Jeong

**Affiliations:** 1 Department of Anesthesiology and Pain Medicine, Chonnam National University Medical School and Hospital, 42 Jebong-ro, Dong-gu, Gwangju, 61469 South Korea; 2 The Brain Korea 21 Project Center for Biomedical Human Resources, Chonnam National University, Gwangju, South Korea

**Keywords:** Carotid endarterectomy, Regional cerebral oxygen saturation, Near-infrared spectroscopy, Propofol, Sevoflurane

## Abstract

**Background:**

The monitoring of regional cerebral oxygen saturation (SrO_2_) using near-infrared spectroscopy is useful method to detect cerebral ischemia during. Sevoflurane and propofol decrease cerebral metabolic rate (CMRO_2_) in a similar manner, but the effects on the cerebral blood flow (CBF) are different. We hypothesized that the effects of sevoflurane and propofol on SrO_2_ were different in patients with deficits of CBF. This study compared the effect of sevoflurane and propofol on SrO_2_ of patients undergoing cerebral endarterectomy (CEA).

**Method:**

Patients undergoing CEA were randomly assigned to the sevoflurane or propofol group (*n* = 74). The experiment was preceded in 2 stages based on carotid artery clamping. The first stage was from induction of anaesthesia to immediately before clamping of the carotid artery, and the second stage was until the end of the operation after clamping of the carotid artery. Oxygen saturation (SrO_2_, SpO_2_), haemodynamic variables (blood pressure, heart rate), respiratory parameters (end-tidal carbon dioxide tension, inspired oxygen tension), concentration of anesthetics, and anesthesia depth (bispectral index score) were recorded.

**Results:**

During stage 1 period (before carotid artery clamping), the mean value of the relative changes in SrO_2_ was higher (*P* = 0.033) and the maximal decrease in SrO_2_ was lower in the sevoflurane group compared with the propofol group (*P* = 0.019) in the contralateral (normal) site. However, there is no difference in ipsilateral site (affected site). SrO_2_ decreased after carotid artery clamping and increased after declamping, but the difference was not significant between two groups. Changes in mean arterial blood pressure was lower in sevoflurane group than propofol group after the carotid artery declamping (*P* = 0.048).

**Conclusion:**

Propofol-remifentanil anesthesia was comparable with sevoflurane-remifentanil anesthesia in an aspect of preserving the SrO_2_ in patients undergoing carotid endarterectomy.

**Trial registration:**

Clinical Trials.gov identifier: NCT02609087, retrospectively registered on November 18, 2015.

## Background

Carotid endarterectomy (CEA) is a useful surgical treatment for reducing the cerebral infarct risk in patients with carotid artery stenosis [1]. Ischaemic brain injury is one of the most severe complications of CEA, caused by cerebral hypoperfusion during temporary carotid artery clamping [2]. Cerebral hyperperfusion syndrome is also a critical, rare complication [3]. Monitoring of cerebral blood flow is essential to prevent these complications; however, continuous monitoring throughout the entire period of CEA is not easy [4].

Monitoring of the regional cerebral oxygen saturation (SrO_2_) using near-infrared spectroscopy (NIRS) is a non-invasive method to observe the oxygen balance of the brain. SrO_2_ determined by arterial oxygen saturation, cerebral blood flow (CBF), haemoglobin and cerebral metabolic rate (CMRO_2_) reflect the balance of oxygen supply and demand of brain tissue [5]. Previous studies indicated that it is possible to detect the changes in CBF and predict the occurrence of cerebral ischaemia by monitoring of SrO_2_ [5–8]. Furthermore, monitoring of SrO_2_ using NIRS is suggested to be a useful method to detect brain ischaemia during CEA [7].

Sevoflurane and propofol are common agents used to maintain general anaesthesia in CEA. Both agents decrease the CMRO_2_ in a similar manner, but the effects on the CBF are different. CBF changes in parallel to the CMRO_2_—a decrease in CMRO_2_ is accompanied by a decrease in CBF, and vice versa. However, sevoflurane per se has a vasodilatory effect and increases the CBF at concentrations > 1 MAC (minimal alveolar concentration). Thus, CBF is abundant compared with CMRO_2_ [9, 10]. Meanwhile, propofol has no vasodilatory effect; it decreases the CBF as CMRO_2_ is reduced. [9] Moreover, the reduction of CBF exceeds that of CMRO_2_ by propofol, resulting in a decrease in the CBF/CMRO_2_ ratio [11, 12]. There have been several reports that the brain oxygen balance is well maintained in sevoflurane anaesthesia compared with that induced by propofol [13–15].

Recent studies measuring cerebral oxygen saturation using NIRS showed higher oxygen saturation in sevoflurane anaesthesia than that induced by propofol [16, 17], suggesting that sevoflurane is better than propofol to maintain the cerebral oxygen balance. However, in these studies, the patients had neither cerebral vascular disease nor deficit of CBF, and the CBF was preserved with both agents from the viewpoint of clinical safety.

In the present study, we hypothesized that the effects of sevoflurane and propofol on SrO_2_ in patients undergoing CEA with deficits of CBF, were different compared to those without any CBF deficits. The primary outcome of the study was the comparison of the effects of sevoflurane and propofol on SrO_2_ in patients undergoing CEA with deficits of CBF. Secondary outcome was the occurrence of any neurologic or hemodynamic deterioration.

## Methods

This prospective randomized controlled trial was approved by the institutional ethics committee of our institution (CNUH-2015-159) and registered in the ClinicalTrials.gov (NCT02609087). After receiving the written informed consent, 74 patients (age, 19–80 years) scheduled to undergo CEA under general anaesthesia were randomly allocated into the sevoflurane group or propofol group by a pre-assembled list generated at random using Excel program. The exclusion criteria were patients with American Society of Anesthesiologists (ASA) physical status ≥4 with low cardiac function, anemia, end-stage renal disease, patients who were low oxygen saturation with pulmonary disease, both carotid artery stenosis, and other neurological disease not related to carotid artery disease. This study was conducted in accordance with the Consolidated Standards of Reporting Trials (CONSORT) 2010 statement [18].

### General management

Patients were given triazolam (Halcion®; Pfizer Korea, Seoul, Korea) for anxiolysis 1 h before arriving in the operating room. After arrival in the operating room, 5-lead electrocardiography, pulse oximetry, and non-invasive automatic blood pressure cuff and end-tidal capnography were implemented for basic monitoring. To assess the depth of anaesthesia, BIS® A-2000™ (Aspect Medical Systems, Natick, MA, USA) was attached to the forehead, and an INVOS®5100B cerebral oximeter using NIRS (Somanetics, Troy, MI, USA) was applied for monitoring of SrO_2_. The two probes were placed at the forehead just above the bispectral index score (BIS) probe facing the medial margin of the other. For continuous blood pressure monitoring (Deltran; Utah Medical Products, Midvale, UT, USA) and to obtain samples for arterial blood gas analysis, a 20-gauge catheter was placed in the radial artery after local anaesthetic infiltration. TOF-watch®SX (Organon Ireland Ltd., Swords, Co. Dublin, Ireland) was used to measure the acceleration of the adductor pollicis muscle as a means of monitoring proper muscle relaxation.

In the sevoflurane group, general anaesthesia was induced by infusion of remifentanil (Ultiva®; GlaxoSmithKline Manufacturing; San Polo di Torrile, Italy) using a target-controlled infusion (TCI) pump (Orchestra Base Primea®; Fresinius, Brezins, France) at a target concentration of 3 ng/dL with bolus injection of 1.5 mg/kg of propofol (1% Anepol®; Hana Pharmaceutical Co., Seoul, South Korea). After confirmation of the loss of consciousness by testing the eyelid reflex, 0.6 mg/kg of rocuronium bromide (Esmeron®; MSD, Kenilworth, NJ, USA) was given for muscle relaxation. A facemask and manual ventilation were applied until adequate sedation (BIS < 60) and muscle relaxation (TOF count 0) were achieved. Endotracheal intubation was then performed. General anaesthesia was maintained with 50% O_2_ at 3 L/min with sevoflurane (Sevofran®; Hana Pharmaceutical Co.), with adjustment of the sevoflurane concentration to maintain a BIS value of 40–50. In the propofol group, anaesthesia induction and maintenance were performed by continuous infusion and changing the concentration of propofol (Fresofol MCT®; Fresinius Kabi, Granz, Austria) with remifentanil at a concentration of 3 ng/dL using a TCI pump. After endotracheal intubation, controlled ventilation was established at a tidal volume of 7 mL/kg and rate of 12/min with FiO_2_ = 0.5, and end-tidal carbon dioxide (ETCO_2_) was maintained between 35 and 40 mmHg by respiratory rate control.

If mean arterial blood pressure (MAP) decreased by < 55 mmHg or was ≥25% of the baseline value, or the systolic arterial pressure dropped below 90 mmHg [19], 100 μg of phenylephrine was injected for correction. Dopamine was infused when hypotension was sustained despite proper anaesthetic depth or if an inotropic agent was required after vessel clamping. If the operator requested, 12.5–25 mg of diluted indocyanine green (Indocyanine Green®; Dongin-Dang Pharmaceuticals; Seoul, Korea) was added. At the end of the operation, total infused fluid volume, blood loss, urine output, anaesthesia time, operation time, and vessel clamping time were assessed.

### Cerebral oxygen saturation and haemodynamics record

This study was divided into two stages. The first stage was from induction of anaesthesia to immediately before carotid artery clamping, and the second stage was from carotid artery clamping to the end of surgery. Before induction of anaesthesia, the basal levels of blood pressure, heart rate, oxygen saturation, BIS, and SrO_2_ in room air were recorded. When haemodynamic stability was established with mechanical ventilation (FiO_2_ = 0.5) after induction of anaesthesia, blood pressure, heart rate (HR), oxygen saturation, BIS and SrO_2_ were recorded (post-induction baseline). At 1-min intervals, blood pressure, HR, oxygen saturation, BIS, SrO_2_, ET_CO2_, anaesthetic concentrations, type and dosage of inotropic agent were assessed after skin incision for 10 min. These values were then recorded every 5 min before carotid artery clamping. After clamping and declamping of the carotid artery, the values were recorded every 1 min for 10 min, including maximal decrease after carotid artery clamping and maximal increase after declamping of SrO_2_. The recording interval was then returned to 5 min.

### Statistical analysis

Previous studies in patients free of cerebrovascular disease showed maximal decreases in SrO_2_ of 9.6% ± 7.7% in the sevoflurane group and 4.2% ± 7.0% in the propofol group. Student’s *t*-test (2-tail) was performed using the G-power program to calculate the sample size showing the difference of maximal decrease between the two groups (effect size = 0.7338623) and 34 patients per group were required to achieve adequate power (*P* = 0.05; β = 0.8) to detect a difference. Considering possible dropout, 37 patients were enrolled per group. Patients’ characteristics and intraoperative variables were analysed using Student’s *t-*test or Pearson’s chi-square test. Changes in SrO_2_ were analysed using Student’s t-test. Continuous variables, including SrO_2_, BIS, blood pressure and heart rate, were analysed by repeated measures analysis of variance (ANOVA) with time as a within-subject repeated measure and the group as a between-subject variable. Statistical analysis was performed using the IBM SPSS Statistics version 20 statistical package (IBM, Chicago, IL, USA). All data are expressed as means ± standard deviation. In all analyses, *P* < 0.05 was taken to indicate statistical significance.

## Results

**Fig. 1:**
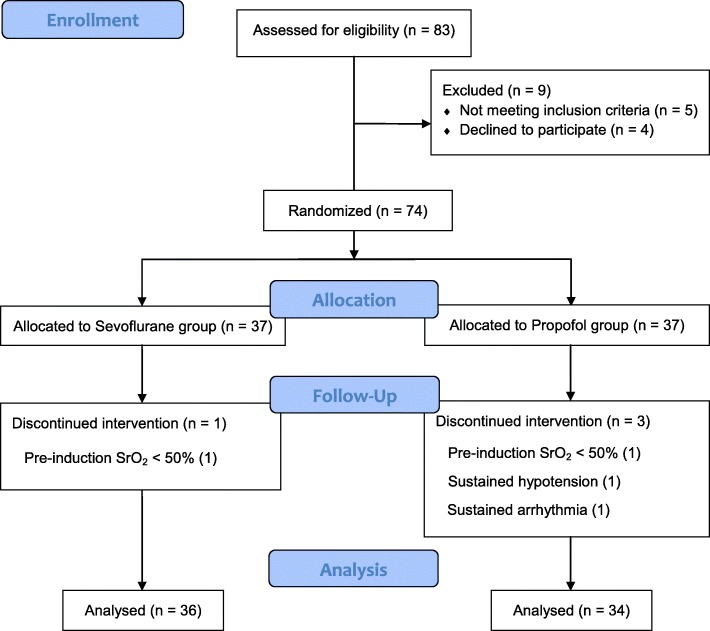
CONSORT flowchart showing the flow of patients through the trial

Among the 74 patients initially included in the study, 2 patients (1 in the sevoflurane group and 1 in the propofol group) with low SrO_2_ (pre-induction basal value < 50%) and 2 patients in the propofol group that showed sustained hypotension or arrhythmia intraoperatively were excluded (Fig. 1). Patients’ characteristics including gender, weight, and height, were similar in both groups. There were no significant differences in intraoperative total fluid administration, blood loss, or urine output between the groups (Table 1). There were no significant differences in preoperative values of SrO_2_, arterial blood pressure, peripheral arterial oxygen saturation, or heart rate between the groups (Table 2). The mean concentrations of intraoperative anaesthetic agents were 1.36 ± 0.19 Vol% for sevoflurane and 2.53 ± 0.37 μg/mL for propofol. The mean remifentanil concentrations were 2.16 ± 0.62 ng/mL in the sevoflurane group and 2.87 ± 1.0 ng/mL in the propofol group; the difference was not significant.

**Table 1:** Patients Characteristics and Intraoperative Variables

	Sevoflurane (*n* = 36)	Propofol (*n* = 34)	*P* value
Sex (Male/Female)	31/5	27/6	0.562
Age (yrs)	69 ± 8	67 ± 7	0.212
Height (cm)	163 ± 7	164 ± 7	0.322
Weight (kg)	64 ± 10	66 ± 10	0.513
Hemoglobin (g/dL)	12 ± 1	13 ± 1	0.273
Underlying diseases			
Hypertension	30	24	0.413
DM	13	15	0.389
Total anesthesia time	181 ± 29	174 ± 26	0.914
Total operation time	161 ± 19	158 ± 17	0.806
Artery clamping time	39 ± 8	38 ± 10	0.401
Fluid administered (mL)			
Crystalloid	1412 ± 377	1618 ± 441	0.069
Colloid	338 ± 218	256 ± 237	0.201
Bleeding (mL)	90 ± 42	116 ± 72	0.159
Urine (mL)	577 ± 279	762 ± 400	0.109

Values are mean ± SD or numbers

**Table 2:** Baseline hemodynamic and oximetry values

	Sevoflurane (*n* = 36)	Propofol (*n* = 34)	*P* value
SrO_2_ (%)-Ipsilateral	64.2 ± 5.7	66.0 ± 5.9	0.074
SrO_2_ (%)-Contralateral	64.1 ± 7.6	67.4 ± 9.2	0.096
SpO_2_ (%)	97 ± 2	97 ± 2	0.407
Mean arterial pressure (mmHg)	103 ± 16	110 ± 20	0.092
Heart rate (beats/min)	66 ± 12	70 ± 17	0.222

Values are mean ± SD. SrO_2_ = regional cerebral oxygen saturation; SpO_2_ = peripheral arterial oxygen saturation

### Changes from the pre-induction period until carotid artery clamping

**Fig. 2:**
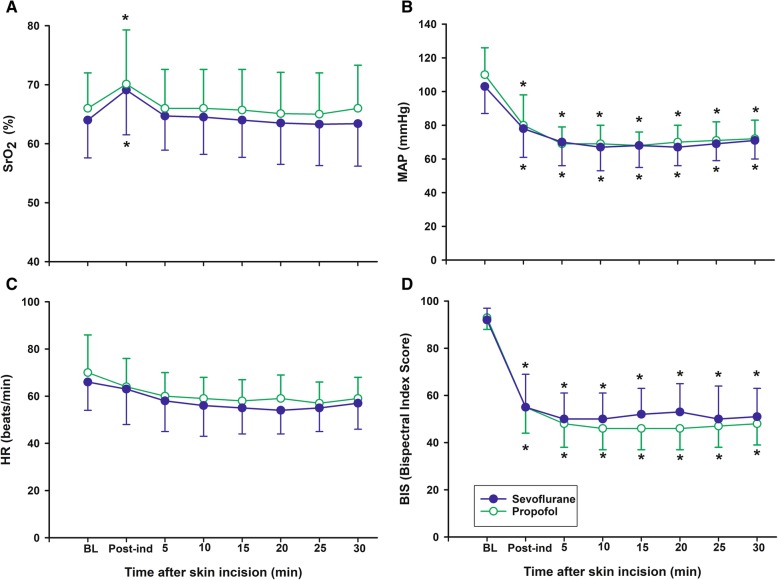
Changes from the pre-induction period until carotid artery clamping. Changes in (**a**) regional cerebral oxygen saturation (SrO_2_), (**b**) mean arterial pressure (MAP), (**c**) heart rate (HR), and (**d**) Bispectral index score (BIS) before carotid artery clamping. BL = baseline before anesthesia induction; Post-ind = post-induction. Groups: Sevoflurane = anesthesia with sevoflurane and remifentanil; Propofol = anesthesia with propofol and remifentanil. Data are expressed as mean ± standard deviation. *P < 0.05 vs Baseline in each group

SrO_2_ increased significantly in both groups after induction of anaesthesia compared with baseline (*P* < 0.001 in the sevoflurane group and *P* = 0.012 in the propofol group). However, there was no difference in the change in SrO_2_ between the groups (Fig. 2a). The mean value of the relative change in SrO_2_ was higher (*P* = 0.033) and the maximal decrease in SrO_2_ was lower in the sevoflurane group compared with the propofol group (*P* = 0.019) in the contralateral site. However, there is no difference in ipsilateral site (affected site) (Table 3). MAP, HR and BIS were similar from induction until 30 min after skin incision, and there were no differences between the groups (Fig. 2).

**Table 3:** SrO_2_ Values in before arterial clamping

	Sevoflurane (*n* = 36)	Propofol (*n* = 34)	*P*-value
Ipsilateral			
Mean SrO_2_ (%)	63.9 ± 6.6	65.3 ± 6.4	0.452
Minimum SrO_2_ (%)	61.8 ± 6.6	63.1 ± 7.1	0.407
Mean value of relative changes in SrO_2_			
compare to baseline (%)	1.3 ± 8.0	**−** 1.6 ± 8.0	0.237
Relative maximum decrease in SrO_2_ (%)			
compare to baseline	3.3 ± 8.8	4.8 ± 9.7	0.504
compare to post-induction	9.9 ± 6.2	8.1 ± 6.2	0.250
Contralateral			
Mean SrO_2_ (%)	67.1 ± 7.5	67.3 ± 6.9	0.452
Minimum SrO_2_ (%)	66.3 ± 8.0	66.3 ± 7.1	0.992
Mean value of relative changes in SrO_2_			
compare to baseline (%)	9.1 ± 20	−1.9 ± 22	0.033
Relative maximum decrease in SrO_2_ (%)			
compare to baseline	−3.9 ± 10.1	0.7 ± 10.5	0.019
compare to post-induction	4.4 ± 3.7	6.6 ± 5.1	0.038

Values are mean ± SD. SrO_2_ = regional cerebral oxygen saturation

### Changes from cardioid artery clamping until the end of surgery

**Fig. 3:**
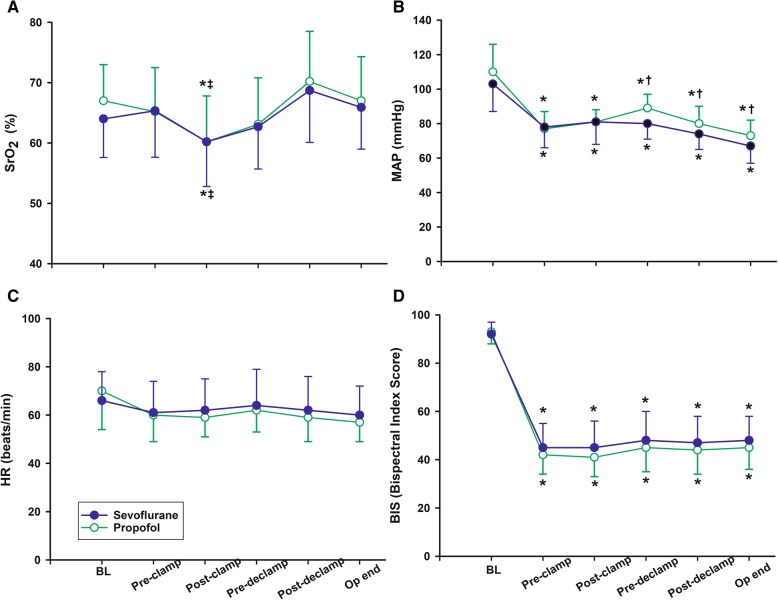
Changes from carotid artery clamping until the end of surgery. Changes in (**a**) regional cerebral oxygen saturation (SrO_2_), (**b**) mean arterial pressure (MAP), (**c**) heart rate (HR), and (**d**) Bispectral index score (BIS) from cardioid artery clamping until the end of surgery. BL = baseline before anesthesia induction; Pre-clamp = immediate before carotid artery clamping; Post-clamp = maximum decrease in SrO_2_ after artery clamping; Pre-declamp = immediate before declamping the artery; post-declamp = maximum increase in SrO_2_ after artery clamping; Op end = operation end. Groups: Sevoflurane = anesthesia with sevoflurane and remifentanil; Propofol = anesthesia with propofol and remifentanil. Data are expressed as mean ± standard deviation. *P < 0.05 vs Baseline, ‡*P* < 0.05 vs Pre-clamp in each group. †*P* < 0.05 vs Sevoflurane group

SrO_2_ decreased significantly after carotid artery clamping in both groups (*P* < 0.001), but there was no difference between the two groups (7.6% ± 4.8% in the sevoflurane group vs. 7.6% ± 5.3% in the propofol group, *P* = 0.857) (Fig. 3a). After carotid artery declamping, SrO_2_ increased significantly, but there was no difference between the two groups (10.0% ± 11.3% in the sevoflurane group vs. 11.8% ± 10.2% in the propofol group, *P* = 0.473) (Fig. 3a). The change in MAP was significantly lower in the sevoflurane group than the propofol group after declamping of the carotid artery (*P* = 0.048) (Fig. 3b). There was no difference in heart rate and BIS score between groups (Fig. 3c and d).

SrO_2_ decreased by > 20% of baseline during surgery in one patient in the sevoflurane group and in five patients in the propofol group, but the differences between groups were not statistically significant (*P* = 0.06). The SrO_2_ of five patients (three in the sevoflurane group and two in the propofol group) fell below 50%. In the pre- and post-carotid artery periods, SrO_2_ decreased by > 20% in two patients. Mild stroke occurred in two patients at postoperative follow-up (one in the propofol group and one in the sevoflurane group), and the SrO_2_ decreases in each patient were assessed: 18% of basal level and 26% of pre-clamping period in a patient in the propofol group; 11% of basal level and 18% of pre-clamping level in a patient in the sevoflurane group. SrO_2_ increased sharply, > 20% of basal level, after reperfusion in one patient in the sevoflurane group; however, no neurological disorders were found.

Continuous infusion of dopamine was required for maintenance of MAP in the majority of patients, and there were no differences in dopamine dosage or vasopressor requirements.

## Discussion

Regional cerebral oximetry using NIRS is a non-invasive method to evaluate the balance between cerebral oxygen supply and cerebral tissue oxygen demand [20]. SrO_2_ indicates the percentage of oxygenated and deoxygenated haemoglobin detected by near-infrared irradiation at two different wavelengths passing through the skull. The near-infrared irradiation used for NIRS shows high penetrability through the scalp and skull, and therefore can reach deep brain tissue for cerebral oxygen saturation measurements of tissue layers including arterial blood, venous blood, and frontal lobe tissue supplied by the anterior cerebral artery and middle cerebral artery. NIRS is a convenient, rapid, non-invasive and continuous method that is superior to other monitoring devices; as such, it is commonly used to monitor cerebral ischaemia and infarction during neuro- or cardiac surgery [21] and in high-risk operations [22].

However, there are limitations for NIRS, such as localised reflection of oxygen saturation only in focal areas and large interindividual differences due to extracranial blood flow including the scalp. The numerical data from NIRS cannot be interpreted as simple numbers due to the different reference values in individual patents affected by systemic arterial oxygen saturation, CBF, haemoglobin concentration and cerebral oxygen metabolic rate. Although the limitations restrict the assessment of recorded SrO_2_ as an absolute value, cerebral ischaemia can be detected and prevented by continuous monitoring of SrO_2_ changes. Samra et al. reported that a decrease in SrO_2_ > 20% of the basal level indicates the occurrence of neurological disorder with 80% sensitivity and 82% specificity [23]. Michel et al. reported that NIRS can recognise cerebral ischaemia during carotid artery clamping in CEA patients with 80% sensitivity and 94% specificity, and it is 6.5 min faster than the somatic-evoked potential [24].

In the present study, SrO_2_ increased rapidly with induction and endotracheal intubation until carotid artery clamping, and then slowly returned to baseline while preparing for surgery. This may be accompanied by increased oxygen supply due to ventilation through the endotracheal tube (FiO_2_ = 0.5) after breathing room air (FiO_2_ = 0.21) and decreased cerebral oxygen metabolism due to general anaesthesia. A previous study in healthy volunteers showed a similar pattern of SrO_2_ changes to those seen in the present study [25].

Mean and minimal SrO_2_ were similar between both groups from induction and incision until carotid artery clamping. SrO_2_ was influenced by the basal level (*P* < 0.001), and average values of SrO_2_ change compared with the basal level were not different between the two groups at the ipsilateral side (affected side). However, there was a difference between the two groups at the contralateral side (normal side), with a 9.1% increase in the sevoflurane group and 1.9% decrease in the propofol group, and the maximal decrease after induction was significantly different between the two groups.

The effects of sevoflurane and propofol on CBF are different. Both agents decrease cerebral oxygen metabolic rate by a similar amount and, as a result, oxygen demand is reduced and CBF is also decreased. Sevoflurane decreases CBF at concentrations < 1 MAC (minimum alveolar concentration; sevoflurane = 2 Vol%) but increases CBF at concentrations > 1 MAC [9, 26], which are thought to be due to the cerebral vasodilation effects of sevoflurane and cerebral autoregulation [27, 28]. Meanwhile, propofol decreases CBF in a dose-dependent manner [12, 29].

In the present study, sevoflurane was administered at a concentration < 1 MAC throughout the entire operation time, and the mean sevoflurane concentration was 1.36 vol%. This result suggests that the effect of sevoflurane on change in CBF may be reduced compared with the pre-induction period. However, SrO_2_ was elevated compared with the baseline, which was thought to be due to increased oxygen supply and decreased cerebral oxygen metabolic rate. The mean value of the relative change in SrO_2_ and maximal decrease were significantly higher in the sevoflurane group than the propofol group. This may have been because the decrease in CBF by propofol is more profound than that by sevoflurane, although the decrease in CMRO_2_ was similar between the two agents.

Interestingly, the SrO_2_ change only occurred on the contralateral but not the ipsilateral side, and there was no significant difference on the ipsilateral side in the peri-clamping period between the two groups. This was associated with differences in drug responses between ipsilateral and contralateral sides. Severe internal carotid artery stenosis induces blood–brain barrier injury and impairs the autoregulation to interrupt vasodilation or vasoconstriction on the ipsilateral side. McCulloch et al. reported stump pressure elevation of the clamped ipsilateral carotid artery after switching anaesthetic agent from sevoflurane to propofol in patients with CEA, and they assumed that this was caused by the vasodilation effect of sevoflurane and vasoconstriction effect of propofol [29]. Simultaneously, the middle CBF rate was decreased when anaesthetic agent was changed from sevoflurane to propofol (ipsilateral side, 53 ± 22 → 43 ± 17 cm/s, *n* = 14; contralateral side, 53 ± 19 cm/s → 37 ± 7 cm/s, *n* = 7). In another group, the agent was changed from propofol to sevoflurane, and the internal carotid artery pressure was decreased to a lesser degree when the middle cerebral artery flow rate remained constant (ipsilateral side, 51 ± 22 → 52 ± 25 cm/s; contralateral side, 43 ± 15 → 49 ± 17 cm/s). Although this result was not statistically significant due to the small sample size, the observation suggested that the affected brain vessels tended to show a lower drug response than those on the contralateral side.

The observation that the data difference between both agents was significant only on the contralateral (non-affected) side may have been due to decreased reactivity on the ipsilateral (affected) side in patients with carotid artery stenosis. Further studies are needed to determine the precise reasons for these results, because CBF rate was not measured in our study and SrO_2_ can be influenced by many other factors. After carotid artery clamping in our study, SrO_2_ changes showed no significant differences in both groups. Previous studies indicated that SrO_2_ remained higher with sevoflurane than propofol [25], and sevoflurane was considered to secure a wider safety margin than propofol when CBF was decreased [16, 17].

However, the effects of propofol and sevoflurane on SrO_2_ were not different after carotid artery clamping in the present study. These discrepancies in the results were likely due to differences in the methods used. Previous studies examined SrO_2_ changes in healthy volunteers or patients with global CBF reduction in the head elevation position, whereas only collateral vessels were supplied because a large vessel was clamped and main CBF was blocked in the present study. During the vessel clamping period, SrO_2_ was decreased by 7.5% compared with the pre-clamping period in both groups in the present study. These observations indicated that cerebral blood supply from the collateral artery could not satisfy the cerebral demand resulting in increased collateral blood flow to compensate for blockage of the main blood flow by clamping. This cerebral haemodynamic state offset the reactions of sevoflurane and propofol to CBF, or blunted the vessel reactivity to both agents.

Another consideration of our study is the effect of intraoperative shunt flow during clamping. Intraoperative carotid bypass (shunt) was not conducted in all patients in the present study. Although placement of carotid artery shunt can increase SrO_2_ especially in symptomatic carotid disease patients [30], the optimal threshold of SrO_2_ for selective shunting using NIRS have not been established [31]. Furthermore routine or selective shunting has no evidence for the better neurologic outcome comparing with no shunting [32]. As a result, haemodynamic control for prevention of stroke guided by evoked potential monitoring during clamping is used instead of shunting in our institution.

In the present study, postoperative mild stroke occurred in two patients, one of whom showed a decrease in SrO_2_ > 20% of the basal level (sensitivity, 50%), while decreases of > 20% in SrO_2_ occurred in six patients in the entire study population (positive predictive value, 17%). Sensitivity of 80% was achieved in a previous study in patients undergoing CEA with regional anaesthesia (deep or superficial cervical plexus block) [23], and the results were revised in a large cohort study performed in 549 patients with general anaesthesia showing 30% sensitivity, 98% specificity, and 37% positive predictive value [33]. Our study with 50% sensitivity and 17% positive predictive value in a relatively small cohort should be repeated in larger numbers of patients to achieve greater reliability.

With the exception of two patients in the propofol group, inotropic agents were used to maintain blood pressure within the normal range in other patients in our study population. Haemodynamic instability often occurs in patients with CEA, and impairment of haemodynamic autoregulation by blunted baroreceptor sensitivity due to carotid artery atherosclerosis is common. In addition, advanced age and medications for comorbidities, such as hypertension or diabetes, are associated with haemodynamic instability. Propofol maintained a higher mean arterial pressure than sevoflurane after carotid artery clamping in this study. The benefit of propofol over sevoflurane with regard to haemodynamic stability was not confirmed, despite many previous studies in various clinical settings. Furthermore, in this study, inotropic agents were necessary to maintain stable arterial blood pressure in both groups, and variation of mean arterial blood pressure tended to be uneven. Therefore, we cannot conclude that propofol is a better agent than sevoflurane for providing haemodynamic stability in patients with CEA, and further research is required.

## Conclusions

Propofol-remifentanil anesthesia was comparable with sevoflurane-remifentanil anesthesia in an aspect of preserving the SrO_2_ in patients undergoing carotid endarterectomy.

## Data Availability

The datasets used and analysed during the current study are available from the corresponding author on reasonable request.

## Abbreviations

BIS: Bispectral index score
CBF: Cerebral blood flow
CEA: Carotid endarterectomy
CMRO_2_: Cerebral metabolic rate
HR: Herat rate
MAP: Mean arterial pressure
NIRS: Near-infrared spectroscopy
SrO_2_: Regional cerebral oxygen saturation
